# Brief Review of Endometriosis and the Role of Trace Elements

**DOI:** 10.3390/ijms222011098

**Published:** 2021-10-14

**Authors:** Ida Osuchowska-Grochowska, Eliza Blicharska, Marek Gogacz, Agata Nogalska, Izabela Winkler, Agnieszka Szopa, Halina Ekiert, Barbara Tymczyna-Borowicz, Mansur Rahnama-Hezavah, Cezary Grochowski

**Affiliations:** 1 Department of Anatomy, Medical University of Lublin, Jaczewskiego 4, 20-090 Lublin, Poland; a.k.nogalska@gmail.com; 2 Department of Analytical Chemistry, Medical University of Lublin, Chodźki 4a, 20-093 Lublin, Poland; bayrena@o2.pl; 3 2nd Department of Gynecology, Lublin Medical University, 20-954 Lublin, Poland; marek.gogacz@umlub.pl; 4 2nd Department of Gynecology, St John’s Center Oncology, 20-090 Lublin, Poland; ikochans@interia.pl; 5 Department of Pharmaceutical Botany, Jagielonian University, Collegium Medicum, 30-688 Kraków, Poland; a.szopa@uj.edu.pl (A.S.); halina.ekiert@uj.edu.pl (H.E.); 6 Department of Conservative Dentistry with Endodontics, Medical University of Lublin, 20-439 Lublin, Poland; barbara.tymczyna@umlub.pl; 7 Department of Dental Surgery Medical University of Lublin, 20-081 Lublin, Poland; mansur.rahnama@umlub.pl; 8 Laboratory of Virtual Man, Medical University of Lublin, 20-439 Lublin, Poland; cezary.grochowski@o2.pl

**Keywords:** endometriosis, trace elements, endometrium, oxidative stress

## Abstract

Endometriosis is a chronic, estrogen-dependent, inflammatory condition that is defined as the presence of endometrial glands and stroma outside the uterine cavity. Despite the progress in research into the mechanisms leading to the development of endometriosis, its cause has not yet been established. It seems to be possible that the formation of oxidative stress may be one of the main causes of the development of endometriosis. There is much research that studies the potential role of trace elements in the appearance of endometrial-like lesions. Most studies focus on assessing the content of selected trace elements in the blood, urine, or peritoneal fluid in women with endometriosis. Meanwhile, little is known about the content of these elements in endometrial-like implants, which may be helpful in developing the theory of endometriosis. Investigations that are more comprehensive are needed to confirm a hypothesis that some trace elements play a role in the pathomechanism of endometriosis.

## 1. The Importance of the Problem

Endometriosis is an enigmatic disease. Rokitansky (1860) was the first to publish the histology of endometriosis. Cullen described both the morphologic and clinical picture (Benagiano and Brosens 2011), while Sampson (1925) provided it the name in use. Ever since, many conflicting theories of pathogenesis have appeared, but none of them fully explain the pathomechanism of the endometriosis entity. For this reason, there are no definitive treatment or prevention measures for this condition [1,2]. It is important to develop new therapeutic options that will treat not only the symptoms of this condition but, above all, its cause [3].

Endometriosis is characterized by the presence of the lining of the womb (endometrium) outside of the uterine cavity. It affects even 15% of women of reproductive age. Endometriosis is suggested to be an autoimmune disease associated with local and systemic immune changes. Moreover, various autoantibodies, including antinuclear and phospholipid antibodies, are involved in the etiopathogenesis of endometriosis [4,5,6,7,8,9,10,11]. Sud et al. (2018) presented a hypothesis in which endometriosis develops on the basis of retrograde menstruation, which causes ectopic endometriotic tissue containing progenitor cells to move through the fallopian tubes to the surface of the ovary into its parenchyma. Therefore, women with a confirmed history of endometriosis have a three-fold higher risk of developing epithelial ovarian cancer, especially clear-cell and endometriotic cancer, which affects approximately 10% of patients with ovarian epithelial neoplasms [12]. Endometriosis was confirmed in 26% of patients with ovarian malignant tumors and in 21% of patients with clear-cell carcinoma, as well as in 6% of women with other ovarian tumors [12]. Moreover, the increasing percentage of cesarean births causes the rising number of patients with endometriosis in the scar after CC, and as a result, the number of patients with clear-cell carcinoma emerging from implant increases [12].

As components of various vitamins and enzymes, trace elements play a key role in many biochemical and physiological processes. Although small amounts of metal ions have protective properties against reactive oxygen species (ROS), their excess can induce cell damage, resulting in various diseases caused by stabilization of abnormal proteins, lipid peroxidation, and oxidation of ROS-scavenging enzymes [13,14].

It is known that ROS takes part in the development of many diseases that are correlated with environmental factors. It has been demonstrated that there is a correlation between ROS in humans and the morbidity of many diseases such as cancers, diabetes, and diseases of the central nervous system.

It seems to be possible that the generation of oxidative stress may be one of the main causes of the development of endometriosis. On the other hand, the inflammatory process accompanying endometriosis may facilitate the implantation and proliferation of ectopic tissue.

In the literature, there are few studies confirming the relationship between the formation of oxidative stress and endometriosis implants. The origin of oxidative stress in the peritoneal cavity in women with endometriosis has not been fully elucidated. It is still unknown if it is caused by higher production of ROS, damage to defense mechanisms, or both of them [15,16]. 

Furthermore, exposure to environmental factors, toxic factors, and toxic heavy metals that lead to an oxidative imbalance in an organism may be a potential initiator of the increased level of ROS.

The literature reports that some metals (known as metalloestrogens), such as lead, mercury, cadmium, or vanadium, may bind to cellular estrogen receptors and then mimic the effects of physiological estrogens. They are related to the etiology of estrogen-dependent diseases, such as breast and endometrial cancer, as well as endometriosis [17,18]. 

Despite the knowledge of many factors that can potentially contribute to the formation of lesions of endometriosis, there is not enough research to explain the role of trace elements in the pathogenesis of this disease.

## 2. Brief Description of Endometriosis

Endometriosis is a chronic, estrogen-dependent, inflammatory condition that is defined as the presence of endometrial glands and stroma outside the uterus. The appearance of endometriosis varies in women and may occur as small peritoneal lesions, advanced forms called endometriomas, which are cysts filled with fluid, most often found in the ovary. Endometriosis can also cause extensive fibrosis and adhesions, which lead to significant distortion of pelvic anatomy and clinical consequences [19,20]. 

Most often, endometrial-like lesions appear in the ovaries, fallopian tubes, peritoneal cavity. However, they may also occur in extra-pelvic locations such as the gastrointestinal tract, lungs, diaphragm, abdomen, or brain.

The endometriotic implants respond to hormones and undergo cyclic growth, differentiation, and shedding. There is an ongoing debate as to whether different forms of endometriosis may have the same mechanism of development.

Clinical presentation of this condition varies in women. The main symptoms are pelvic pain and infertility. Endometriosis is also associated with dysmenorrhea, dyspareunia, and dysuria [21,22,23,24,25]. Pain may appear suddenly and transiently throughout the menstrual cycle, but most often cyclically.

The gold standard diagnosis of endometriosis in the peritoneal cavity is carried out by laparoscopic visualization and histologic examination of lesions. It seems to be an important problem that there is still no reliable clinically diagnostic method or pathognomonic clinical finding, which may allow accurate diagnosis of endometriosis without the need for surgery or histopathologic evaluation [26].

## 3. Epidemiology of Endometriosis

It is estimated that endometriosis occurs in 2–22% of reproductive-aged women in the world [27]. This disease most often affects women at the age of 25–35 years [20]. It rarely occurs in the postmenopausal period and before the menarche. In the literature, there are described isolated cases of endometriosis in men receiving hormone treatment for prostate cancer.

The important problem in the treatment of endometriosis is that the average delay in diagnosis of this disease is almost 6.7 years. It means that even 3–12 years can pass between the appearance of first symptoms and a definitive diagnosis of endometriosis [28]. 

## 4. Theories of Endometriosis

Despite the progress in research into the mechanisms leading to the development of endometriosis, its cause has not yet been established.

The best known and the most widely accepted theory is the one proposed in 1927 by Sampson, based on the finding of the retrograde transport of menstrual blood through the fallopian tubes into the peritoneal cavity and then implantation of displaced endometriotic cells, but it does not allow the exact cause of the disease to be determined. It is not known why only some women develop endometriosis, while retrograde menstruation occurs in most women of childbearing age, and what factors are involved in the etiopathogenesis of this disease [29,30,31]. 

An interesting theory trying to explain the cause of endometriosis development seems to be the theory of immune deficit. Under physiological conditions, endometriotic cells that enter the peritoneal cavity are eliminated by non-specific and specific mechanisms of the immune response that prevent the development of endometriosis [7]. A properly functioning immune system in the peritoneal cavity protects over 85% of women against the development of endometriosis [32,33].

The greatest changes are mainly observed in the peritoneal cavity, while the peritoneal fluid, which provides a specific environment for the pelvic organs, can facilitate the implantation of endometriotic cells that move along with the retrograde menstrual flow. At the same time, the active substances contained in the fluid may be responsible for the metaplasia of the peritoneal cells to the glandular cells and the stroma of the endometrium. Therefore, the composition of the peritoneal fluid that contacts the endometriotic lesions may reflect changes in the course of the disease [34]. 

Another well-known theory is the implantation theory, the spread of endometriosis directly. The transfer of the endometrium outside the uterine cavity may occur during cesarean delivery, in the case of laparoscopic surgery, and after the perineal incision [3,35,36,37]. 

Metaplasia of the cells in the lining of the peritoneum and, in rare cases, dissemination may also contribute to the formation of endometriotic implants. Endometriosis can be spread through blood and lymph vessels to distant organs (lungs, brain, bones) [38]. 

In the literature, there are few studies confirming the relationship between the formation of oxidative stress and endometriosis implants. There are several cellular pathways through which oxidative stress can lead to the formation of endometriotic lesions. Oxidative stress and the presence of excess ROS can damage tissue, disregulate protein activity, gene expression, and induce rapid cellular division [39,40,41,42,43].

The role of autophagy was underlined in several papers, which is induced in stromal and glandular epithelial cells throughout menstruation. Hyperplasia of endometriotic cells is driven by aberrant autophagy, which is also upregulated by hypoxia. Entometriotic tissue usually occurs in anoxic conditions, which promotes autophagic processes and ROS production [44].

Many risk factors for endometriosis have been described in the literature. Factors that are associated with increased risk for this disease are: earlier age at menarche, shorter menstrual cycle length, taller height, alcohol use, caffeine intake. Incidence of laparoscopically confirmed endometriosis by demographic, anthropometric, and lifestyle factors [45,46,47,48,49,50,51,52]. 

Oxidative stress activity and ROS are reported to be increased in people with endometriosis. Factors that may decrease the risk for endometriosis are: parity, current oral contraceptive use, smoking, higher body mass index, regular exercise, eating fish and omega 3 fatty acids [47,48,53,54,55,56]. 

Despite the knowledge of many factors that can potentially contribute to the formation of lesions of endometriosis, there is not enough research to explain the role of trace elements in the pathogenesis of this disease [57]. 

## 5. Diagnosis of Endometriosis

Endometriosis is an insidious disease, often misdiagnosed and confused with other conditions. It negatively affects the quality of women’s life. Early diagnosis is most important in the treatment of endometriosis. Currently, the time that passes from the first symptoms of the disease to proper diagnosis is seven to ten years.

Endometriosis is a difficult disease to diagnose for several reasons. One of the potential factors is presumably a lack of understanding of endometriosis due to its heterogeneity by healthcare professionals. A painful clinical presentation is not synonymous and pathognomic with endometriosis. The pain can be presented in many forms, such as dysmenorrhea, dyspareunia, or chronic pelvic pain. Moreover, the pain can be associated with urinary and/or digestive symptoms, which may complicate the diagnosis [22,58]. Due to the different forms of endometriosis, unspecific symptoms, and variation of levels of hormones during the menstrual cycle, it is difficult to standardize the method that would be suitable for all patients.

Diagnosis of endometriosis should be based first of all on patient interviews, pelvic examination, and imaging. The common problem in the diagnostic process is the lack of noninvasive tests, such as sensitive serum markers, which causes a delay in the differential diagnosis.

Although endometriosis cannot be ruled out during standard physical examination [59], there are several abnormalities, which are available to detect during clinical examination, such as pelvic pain upon mobilization thickened area involving the uterosacral ligament, the upper third of the posterior vaginal wall, or the torus uterinus, palpable sensitive nodules as well as visible bluish lesions on the vaginal fornix [60]. Moreover, physical examination during menstruation can improve detection. Therefore, there is a great need for noninvasive imaging methods development, which could detect the location extensiveness of endometriotic lesions [61]. 

There are several key factors detected during physical examination, such as vaginal palpation, together with speculum examination, which allow visualizing deep infiltrating endometriosis.

Moreover, tenderness of the uterus, as well as the pelvis, uterus fixation, and mobility should be assessed.

Laparoscopic visual inspection with histopathological confirmation is considered the gold standard for the diagnosis of endometriosis. The pathologies observed during the laparoscopic exam are described as endometriomas, peritoneal implants, deep infiltrating nodules of endometriosis, and peritoneal windows. There is a great variety in morphology, color, and size of endometriotic implants among the population [62,63,64]. 

At least two histological features are required to confirm endometriosis, listed as: endometrial glands, endometrial epithelium, hemosiderin-laden macrophages, and endometrial stroma.

Visual diagnosis was proved to be unreliable in several studies. It turned out that only 54–67% of cases of visually assessed endometriotic lesions had histological confirmation.

Lesions suspected of being endometriotic implants may turn out as inflammatory changes, foreign body reactions, hemangiomas, mesothelial hyperplasia, and hemosiderin deposits rather than endometriosis.

Current imaging techniques do not allow accurate staging of endometriosis as they lack the resolution necessary to visualize superficial implants and small ovarian endometriomas and cannot detect the presence, type, or extent of endometriotic adhesions [65]. 

An ultrasound examination is a diagnostic tool, allowing the explanation of underlying symptoms of endometriosis as well as locating the pathological alterations of the endometrium and its severity. Several ultrasound techniques have been established; however, none has been validated yet.

For instance, transvaginal sonography has been found to be the finest technique in the diagnostic process of deep infiltrating endometriosis as well as pelvic endometriosis [66]. 

Moreover, several authors reported the ability of ultrasound examination in the detection of bowel and non-bowel deep infiltrating endometriosis and ovarian endometriosis [67].

Ultrasound examination such as transvaginal sonography was also found useful in planning surgical treatment in the pouch of Douglas obliteration and severe deep infiltrating endometriosis [68,69,70].

The main flaw of currently used imaging techniques is their lack of resolution, allowing proper visualization of several endometrial pathologies such as small ovarian endometriomas and superficial implants. Moreover, the assessment of the type and extent of endometriosis pathologies is valid.

Magnetic resonance imaging and transvaginal sonography allow the visualization of retroperitoneal space and, more importantly, the extension and presence of deep pelvic endometriosis, including bowel and non-bowel factors.

Despite the common accessibility to transvaginal sonography, one has to remember that this technique only allows the visualization of endometriotic cysts [71]. 

It is estimated that the sensitivity of a clinical trial in the diagnosis of endometriosis is about 25% in the case of vaginal-rectal septal endometriosis and only 3–5% in the case of Douglas sinus and urinary bladder endometriosis. Ultrasound examination is more sensitive in detecting endometriotic cysts, but the sensitivity of this examination in relation to other forms of the disease is only slightly higher [72]. It has also been proven that ultrasonography is a sensitive method of detecting endometriosis in the rectal wall, but a very important limitation of this method is the experience of the examiner. It is postulated that this method should only be used in reference centers with extensive diagnostic experience. In conclusion, it should be remembered that the ultrasound examination is not sensitive enough to rule out endometriosis. Very often, a negative result of TV ultrasound examination is the basis for excluding pathologies in the reproductive organ, which in turn delays the proper diagnosis and treatment.

Ultrasound examination of deeply infiltrating endometriosis with the use of gel contrast is a specialized ultrasound examination carried out with a vaginal probe. According to the recommendations of the International Deep Endometriosis Analysis (IDEA) Group, the technique of endometriosis ultrasound examination should include four basic steps: Basic measurements, evaluation of the ovaries, uterus, endometriotic cysts, and adenomyosis (i.e., endometriotic lesions inside the uterine muscle membrane). This is the stage where most standard ultrasound examinations in gynecology end. Assessment of the “soft markers” of endometriosis, i.e., the assessment of ovarian and uterine mobility, possible adhesions, or hydrocele is the “sliding sign” [65,68,69,70,73].

### Biomarkers in Endometriosis

For many years, many laboratory tests have been carried out to identify markers in blood serum, endometrium, and peritoneal fluid that would allow for early and noninvasive diagnosis of endometriosis. So far, it has not been found that any of the biochemical parameters meet the criteria of screening or diagnostic testing in endometriosis [74,75]. 

Serum markers such as cytokines, adhesion molecules, markers of angiogenesis or inflammation are sought for their use in the diagnosis of endometriosis. To date, none of the investigated markers is specific for endometriosis because many of them are present in other gynecological conditions [76].

It is assumed that a noninvasive method of diagnosis of endometriosis should be a peritoneal marker that is present in the endometrium. The tissue can be easily collected by endometrial biopsy without elongated anesthesia. It has been proven that changes in the expression of genes and proteins typical of ectopic foci diseases can also be found in the cavity endometrium uterus [77]. 

In literature, many potential markers have been investigated, but due to the hormone cycle and variation in the amount of peritoneal fluid, it is difficult to standardize the method [78]. 

One of the more widely used biomarkers in endometriosis is the CA-125 protein. However, it should be strongly emphasized that this parameter is not specific to endometriosis; its increased values characterize various clinical conditions, such as menstruation, pregnancy, neoplastic processes, sarcoidosis, peritoneal inflammatory processes, pericarditis, circulatory failure, liver diseases, diabetes mellitus, and connective tissue diseases.

Other factors that may be potential markers in the diagnosis of endometriosis are interleukin 8, vascular endothelial growth factor, platelet-derived growth factor, and nerve growth factor.

It was evaluated that levels of mRNA and protein IL-8 were elevated in the endometrium of patients suffering from endometriosis, compared with healthy women.

The vascular endothelial growth factor is one of the most significant factors that take part in angiogenesis. It is suggested that VEGF plays an important role in the development of endometriosis due to the elevated level of VEGF in a late secretory phase of the menstrual cycle in women with endometriosis.

The role of nerve growth factor GF is known in the pathogenesis of ovarian and breast cancer and presumably in polycystic ovarian syndrome. The main symptom of endometriosis that affects 60% of women with endometriosis is pain, wherefore NGF plays here a significant role in the development and sustentation of the disease [79,80,81,82,83]

Despite the knowledge of many factors that can potentially lead to the formation of endometrial-like lesions, so far, it has not been found that any of the biochemical parameters meet the criteria of screening or diagnostic testing in endometriosis. Few studies are available in the literature, and they are critically evaluated to determine whether long-term exposure to metalloestrogens is a significant cause of the development of endometriosis. A greater tendency for the accumulation of toxic heavy metals within the pathologically altered endometrium and metalloestrogens may be important etiological factors of estrogen-dependent diseases in women such as endometriosis.

It seems not only a topical issue but also results from the need to determine the basis of many endometriotic diseases, which may be related to the level of certain trace elements.

## 6. Trace Elements and Endometriosis

Heavy metals are highly present in the environment. Cadmium compounds are present in stabilizers, fertilizers, production of batteries as well as plastic. The exposure may act through respiratory (smoking) as well as digestive system [84,85]. Human exposure to lead was highly visible in plumbers during radiator repairing, renovating old buildings, metal and construction working as well as welding.

Lead and cadmium were proven to induce the lipid per oxidation as well as the formation of ROS, which interferes with many antioxidant defense enzymes such as catalase, glutathione peroxidase, super-oxidase [86]. This generates an imbalance between the production and removal of ROS in cells, causing damage to DNA as well as proteins; however, its role in endometriosis still remains unknown.

Cobalt (II) and chromium (III) show cytotoxic properties. These metals have a destructive effect on the cell membrane, lysosomes, and mitochondria, which leads to disorders of cell metabolism [87,88]. 

There are only a few studies analyzing the role of cadmium in endometriosis, and among six studies, only one found a positive association between cadmium presence and endometriosis [89].

A study performed by Kim proved that co-exposure to cadmium as well as lead was associated with a higher incidence of hospitalization in patients suffering from endometriosis [90].

The contamination with mercury occurred mostly through dental treatment, recycling of fluorescent lamps as well as gold mining [91]. 

Yilmaz et al. suggested that the presence of metalloestrogens in endometrial-like lesions, as well as normal endometrium, may influence the development of endometrial polyps [92,93]. 

They proved that, in fact, the heavy metal serum level in patients with endometrial polyps was low; however, the Cu/Zn ratio was increased in those patients [94]. Because of the role of Cu and Zn in the organism, the Cu/Zn ratio is used as a marker of oxidative stress and has been used in diagnostic processes in immune dysfunction, inflammation as well as gynecological tumors [95].

The ratio imbalance was caused by low Zn levels in polyps. Moreover, Ni and Pb levels were also decreased in those patients; however, it was hard to explain the reason by the authors due to the study limitations.

Recently, a systematic review performed by Sirohi et al. analyzed environmental exposures to endocrine-disrupting chemicals and their influence on endometriosis. The authors found a positive correlation between copper as well as chromium and prevalence of endometriosis, however, only in one study. Cadmium, lead, and mercury were not found to be associated with this disease, and there were conflicting results for the correlation with nickel [96].

Hayashi et al. published a paper where they presented the first ovarian endometriosis model in wild-type mice. The analysis of the ovarian endometriotic stroll area revealed vas iron deposits, which may promote endometriosis through the oxidative stress processes. They underlined the role of increased oxidative stress in ovarian follicles and its association with the decreased FSHR expression as well as a decreased number of connected fetuses [97]. Moreover, endometriotic stroll cells were found to have high iron affinity [98]. 

Zinc is suggested to have a key role in anti-inflammatory, antioxidant, and immune regulation processes. Few studies reports decreased levels of Zn in patients with endometriosis, suggesting its role in this disease [99]. Zn is known as an inhibitor of MMPs, and increased levels of MMP-2 and MMP-9 were found in women suffering from endometriosis [94,100]. Moreover, high MMP-2 levels are correlated with advanced endometriosis [101], and few studies describe the clinical effectiveness of zinc supplementation [102,103]. 

Nickel has been recently marked as a risk factor of endometriosis. A study performed by Borghini et al. showed a Ni allergic contact mucositis prevalence of about 90.3% in patients with endometriosis compared to study control. Moreover, the authors administrated a low-Ni diet to those patients for a period of three months, achieving a significant improvement of all symptoms typical of endometriosis [104]. 

It is well known that some metalloestrogens such as cobalt, copper, nickel are essential minerals that are required in trace amounts for physiological human body function. When the amounts of these minerals exceed the amount essential for proper body function, they begin to interfere with the hormone receptors. There are also some metalloestrogens, such as cadmium, aluminum, and lead, which are not useful by the body in any amount.

Some heavy metals can activate multiple signaling pathways, in addition to their effects on signaling pathways; furthermore, they also may induce oxidative stress. There are only a few studies that have assessed the effects of toxic metals on the female genital tract and, in particular, endometrial tissue [105]. Most studies focus on assessing the content of selected trace elements in the blood, urine, or peritoneal fluid in women with endometriosis. Meanwhile, little is known about the content of these elements in endometrial implants, which may be helpful in developing the theory of endometriosis and the use of trace elements or ROS manipulation for therapy of endometriosis.

A study in a group of Sri Lankan women with endometriosis reported higher blood nickel levels as compared to women without this disease. Concentrations of cadmium and lead were similar in both groups [106]. 

In different analyses, a connection is observed between the trace elements such as cadmium, chromium, copper, and endometriosis. It is known that cadmium decreases estrogen levels; therefore, since endometriosis is an estrogen-dependent disease, this finding can be plausible [94,107] in Table 1.

The analysis of several trace elements among Asian women revealed that concentrations of trace elements such as zinc, lead, and cadmium were significantly different in a group of endometriosis patients and the control group. The zinc concentration in the blood was higher in patients with endometriosis than in the control group. Furthermore, the lead levels and cadmium levels were increased in patients with endometriosis. The concentrations of copper, manganese, iron, mercury, and chromium were not significantly different between the two groups [99]. 

A study conducted by Messali et al. showed that blood zinc level in women who suffer from endometriosis is decreased, and it can confirm that this trace element may possibly affect the multifactorial pathogenesis of endometriosis. Zinc is suspected of interfering with many biological processes, such as inflammation and immunity, which seem to be essential in the development of endometrial-like lesions [108]. 

Among infertile Japanese women, the hypothesis was presented that higher cadmium exposure is associated with endometriosis. Cadmium may act like estrogen and be a potential risk factor for estrogen-related diseases such as breast cancer and endometriosis. However, the research did not show an association between the concentration of cadmium in urine and endometriosis [109]. 

**Figure 1:**
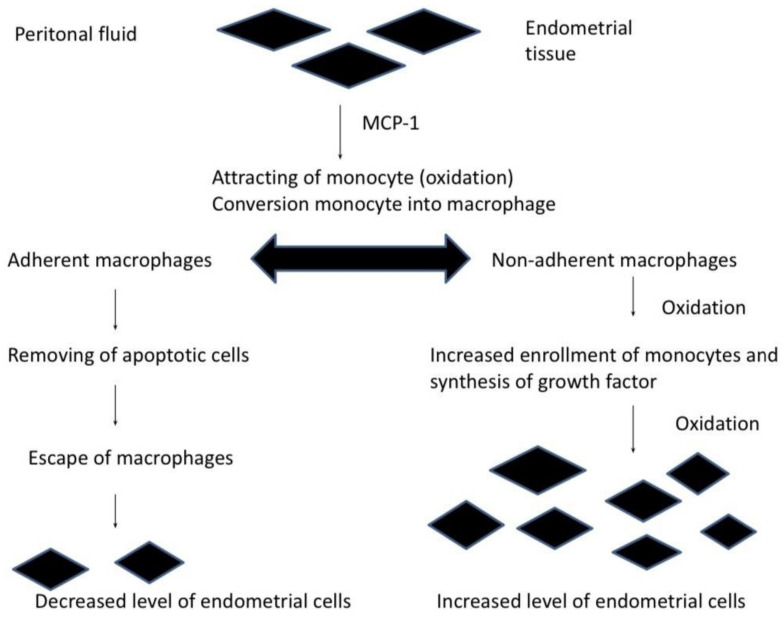
Oxidative stress in the mechanism of endometriosis.

The peritoneal fluid, which has direct contact with endometrial-like lesions, may reflect changes in the course of this disease. In the Polish study, it was shown that disrupted iron homeostasis in the peritoneal cavity of women with endometriosis plays a role in the pathogenesis of this disease. The concentration of iron was higher in peritoneal fluid obtained from women with endometriosis as compared to the control group [110]. In another study, it is suggested that copper (a redox-active metal) appears to be associated with the etiopathogenesis of oxidative stress in endometriosis (Figure 1). The concentration of copper was significantly higher in the patients with endometriosis compared to the control group [111]. 

These data do not support a role for cadmium in the onset of the growth of endometriotic diseases but suggest a possible relationship with lead.

It has been demonstrated that the gastrointestinal absorption of both Pb and Cd increases in women with depleted stores of iron, calcium, and zinc [112,113]. Moreover, important bleedings associated with endometriosis may conceivably contribute to an additional depletion of the iron stores and, in turn, facilitate Cd or Pb absorption [114]. 

In this work, the overall levels of Cd and Pb were lower than reference values in a non-occupationally exposed population in Europe.

In conclusion, these data do not support a role for Cd but suggest a relationship with Pb in the onset of the growth of human endometriosis or deep endometriotic (adenomyotic) nodules of the rectovaginal septum.

## 7. Positive Effects of Trace Elements

Zinc is one of the most crucial elements responsible for the proliferation and differentiation of the reproductive system cells. Moreover, it takes part in the ovulation process, development of spermatozoa, fetal development, physiological pregnancy. Proper zinc levels ensure correct testosterone homeostasis, sperm parameters as well as proper folate cycle [115]. 

Studies suggest the role of trace elements in antioxidant effect among pregnant women through the regulation of hormone and enzyme levels responsible for differentiation of fetal cells and fetal development.

Animal studies report harmful effects of zinc deficiency in women, such as abnormal LH and FSH homeostasis, teratogenic effect, abnormal ovarian development, preeclampsia, and low infant birth weight [104,116]. 

Several trace elements, including iron, gold, selenium, nitrogen, were found to participate in angiogenesis (stabilizing and promoting the generation of new blood vessels). However, excess of selected trace elements such as free iron could result in uncontrolled vasculo and angiogenesis as well as the formation of abnormal vessels by increasing VEGF production.

On the other hand, selenium was found to regulate apoptosis and the cell cycle and is used in cancer therapy. It reduces microvessel formation and angiogenesis by decreasing the VEGF levels of human umbilical vein and arterial cells [117]. 

Copper plays a crucial role in cell division processes. Moreover, it is a part of several enzymes such as superoxide dismutase SOD1 and SOD3, ceruloplasmin, cytochrome c, metallothionein. This particular trace element is also an important factor of fertility process, including gametogenesis, and plays a structural role in testical somatic cells and sperm and prostate liquids. It is responsible for the distribution of androgen and regulation of the hypothalamic pituitary testis line. The improper homeostasis may result in different male fertility abnormalities such as improper production of hormones, sperm levels, etc. [118]. 

Studies reported a positive effect of antioxidant supplementation (vitamin C, vitamin E, selenium, copper, and zinc) in women with confirmed endometriosis. An inverse correlation was noted in patients on an antioxidant diet with the intensity of the disease [119,120]. 

## 8. Trace Elements and Its Role in the Diseases

Despite the fact of the positive zinc effect on the human body, its deficiency was linked with polycystic ovary syndrome. Several studies report the positive effect of zinc supplementation in patients with PCOS. Moreover, mentioned trace element has a substantial impact on the pathogenesis of primary dysmenorrhea through the reduction in prostaglandins, improving the microvascular net of endometrium tissue [121,122,123]. 

Mercury was found to induce oxidative stress processes causing fertility problems in patients exposed to this element. A study performed by El-Badry et al. analyzed that dental staff reported a higher incidence of preeclampsia, spontaneous abortion [104]. Moreover, Vejrup et al. found lowered birth weight in newborns of women exposed to mercury. They also linked irregular menstrual cycles as an effect of exposure. Dysmenorrhea and abdominal pain were also described as symptoms of mercury exposure [124]. Lead was found to have similar fertility negative effects. Sallmén et al. claimed that occupational exposure to lead may lead to pregnancy delay and a higher risk of infertility. Moreover, maternal exposure resulted in delayed growth and pubertal development as well as spontaneous abortion [125].

## 9. Conclusions

There is no doubt that endometriosis leads to deterioration of the quality of life; therefore, there is a great need to develop new therapeutic approaches that will treat not only the symptoms of endometriosis but, above all, its cause. It seems to be an important problem that there is still no reliable clinically diagnostic method or pathognomonic clinical finding, which may allow accurate diagnosis of endometriosis without the need for surgery or histopathologic evaluation. The main reason that does not allow improve diagnostic and treatment of endometriosis is that pathomechanism of endometriosis is not fully known.

It seems to be possible that the formation of oxidative stress may be one of the main causes of the development of endometriosis. There is much research that studies the potential role of trace elements in the appearance of endometrial-like lesions. Most studies focus on assessing the content of selected trace elements in the blood, urine, or peritoneal fluid in women with endometriosis. Meanwhile, little is known about the content of these elements in endometrial-like implants, which may be helpful in developing new or supplementing present theories of endometriosis. 

However, investigations that are more comprehensive are needed to confirm a hypothesis that some trace elements play a role in the pathomechanism of endometriosis.

## Figures and Tables

**Table 1:** Concentration of trace elements in patients with endometriosis.

Trace Element	Effect of Element on the Mechanism of Endometriosis	Concentration Range	Source of Specimen	Number of Patients	Method	References
Antimony	Not available	0.04–0.04 ug/L	Urine		ICP-MS	Pollack et al. (2013)
Arsenic	Not available	4.94–10.83 ug/L				
Barium	Not available	1.18–2.68 ug/L				
Beryllium	Not available	0.01–0.04 ug/L				
Cadmium	Formation of ROS Decreases estrogen levels	0.21–036 ug/L	Blood			
		0.13–0.31 ug/L	Urine			
		0.7–0.9 ug/L	Blood	50	TXRF	Silva et al. 2013
		Mean 2.9 ug/g creatinine	Urine		GFAAS	Heilier et al. (2006)
		Mean 1.9 ug/L	Blood			
		0.8 ug/g creatinine	Urine	59		Heilier et al. (2004)
		0.9 ug/g creatinine		385	-	Buchet et al. (1990)
		0.7 ug/g creatinine		920	-	Sartor et al. (1992)
		0.9 ug/g creatinine		147	-	Staessen et al. (1992)
		0.8 ug/g creatinine		385	-	Hotz et al. (1999)
		0.7 ug/g creatinine		544	-	Jarup et al. (2000)
		0.30–2.49 ug/g creatinine		54	ICP-MS	Hiroaki et al. (2008)
		Mean 0.42 ug/L	Blood	68		Lin-Lai et al. (2017)
Cesium	Not available	3.27–5.70 ug/L	Urine			Pollack et al. (2013)
Chromium	Cytotoxic properties	0.47–1.30 ug/L				
		mean 0.51 ug/L	Blood	68		Lin-Lai et al. (2017)
Cobalt	Cytotoxic properties	0.34–0.64 ug/L	Urine			Pollack et al. (2013)
Copper	Decreases estrogen levels	7.08–13.80 ug/L				
		Mean 1088.00–273.58 ug/mL	Blood	72		Turgut et al. (2013)
		Mean 0.39 ug/L	Blood	68		Lin-Lai et al. (2017)
Iron	Participation in angiogenesis	123–504μg/dl	Peritoneal fluid	50	TIBC	Polak et al. (2010)
		59.8, 111.0 mg/L		57		Langendonckt et al. (2002)
		Mean 1185.86 ug/L	Blood	68	ICP-MS	Lin-Lai et al. (2017)
Lead	Inducing oxidative stress	0.5–0.73 ug/dL				Pollack et al. (2013)
		0.02–0.35 ug/L	Urine			
		8.6–13.3 ug/L	Blood	50	TXRF	Silva et al. (2013)
		Mean 1.7 ug/L			GFAAS	Heilier et al. (2006)
		Mean 13.37 ug/L		68	ICP-MS	Lin-Lai et al. (2017)
Manganesse	Not available	0.96–1.87 ug/L	Urine			Pollack et al. (2013)
		Mean 0.72 ug/L	Blood	68		Lin-Lai et al. (2017)
Mercury	Inducing oxidative stress	0.41–1.04 ug/L				Pollack et al. (2013)
		Mean 0.15 ug/L		68		Lin-Lai et al. (2017)
Molibdenum	Not available	27.05–63.80 ug/L	Urine			Pollack et al. (2013)
Nickel	Interfere with the hormone receptors.	3.07–6.39 ug/L				
		1.9–3.3 ug/L	Blood	50	TXRF	Silva et al. (2013)
Tellurium	Not available	0.04–0.06 ug/L	Urine		ICP-MS	Pollack et al. (2013)
Thallium	Not available	0.1–0.19 ug/L				
Tin	Not available	0.37–0.86 ug/L				
Tungsten	Not available	0.02–0.11 ug/L				
Zinc	Proliferation and differentiation of the reproductive system cells. Key role in anti-inflammatory, antioxidant, and immune regulation processes	160.6–408.4 ug/L				
		Mean 6.72 ug/L	Blood			
